# Gender‐biased kidney damage in mice following exposure to tobacco cigarette smoke: More protection in premenopausal females

**DOI:** 10.14814/phy2.14339

**Published:** 2020-01-24

**Authors:** Abdullah Kaplan, Emna Abidi, Nada J. Habeichi, Rana Ghali, Hiam Alawasi, Christina Fakih, Kazem Zibara, Firas Kobeissy, Ahmad Husari, George W. Booz, Fouad A. Zouein

**Affiliations:** ^1^ Department of Pharmacology and Toxicology Faculty of Medicine American University of Beirut Beirut Lebanon; ^2^ Biology Department Faculty of Sciences‐I Lebanese University Beirut Lebanon; ^3^ Department of Biochemistry and Molecular Genetics Faculty of Medicine American University of Beirut Beirut Lebanon; ^4^ Department of Internal Medicine, Respiratory Diseases and Sleep Medicine American University of Beirut Medical Center Beirut Lebanon; ^5^ Department of Pharmacology and Toxicology University of Mississippi Medical Center Jackson MS USA

**Keywords:** cigarette smoking, gender differences, inflammation, kidney damage, oxidative stress

## Abstract

Multiple clinical studies documented renal damage in chronic cigarette smokers (CS) irrespective of their age and gender. Premenopausal female smokers are known to exert a certain cardiovascular and renal protection with undefined mechanisms. Given the multiple demographic variables within clinical studies, this experimental study was designed to be the first to assess whether gender‐biased CS‐induced kidney damage truly exists between premenopausal female and age‐matched C57Bl6J male mice when compared to their relative control groups. Following 6 weeks of CS exposure, cardiac function, inflammatory marker production, fibrosis formation, total and glomerular ROS levels, and glomerulotubular homeostasis were assessed in both genders. Although both CS‐exposed male and female mice exhibited comparable ROS fold change relative to their respective control groups, CS‐exposed male mice showed a more pronounced fibrotic deposition, inflammation, and glomerulotubular damage profile. However, the protection observed in CS‐exposed female group was not absolute. CS‐exposed female mice exhibited a significant increase in fibrosis, ROS production, and glomerulotubular alteration but with a pronounced anti‐inflammatory profile when compared to their relative control groups. Although both CS‐exposed genders presented with altered glomerulotubular homeostasis, the alteration phenotype between genders was different. CS‐exposed males showed a significant decrease in Bowman's space along with reduced tubular diameter consistent with an endocrinization pattern of chronic tubular atrophy, suggestive of an advanced stage of glomerulotubular damage. CS‐exposed female group, on the other hand, displayed glomerular hypertrophy with a mild tubular dilatation profile suggestive of an early stage of glomerulotubular damage that generally precedes collapse. In conclusion, both genders are prone to CS‐induced kidney damage with pronounced female protection due to a milder damage slope.

## Graphical abstract

Mice of both genders exposed to 6 weeks of tobacco cigarette smoke are prone to kidney damage with pronounced female protection due to a milder damage slope.

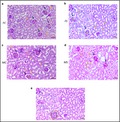

## 1 INTRODUCTION

Chronic kidney disease (CKD) remains a major global health burden with a high rate of morbidity, mortality, and healthcare expenditure (Astor, Hallan, Miller, Yeung, & Coresh, 2008; Grams et al., 2018; Saran et al., 2017; Wen et al., 2008). Global prevalence is estimated to range between 11% and 13%, almost surpassing diabetes, which calls for immediate preventable and interventional strategies to delay the development or progression of CKD (Hill et al., 2016). Patients diagnosed with CKD are not only at high risk of end‐stage renal disease development, but also of multiple comorbidities, including 20‐ to 30‐fold increase in cardiovascular diseases risk. Systemic effects of inflammation, oxidative stress, and uremic toxins accumulation are all involved in the adverse outcomes following CKD diagnosis (Junior et al., 2014; Yoon et al., 2009). Since CKD occurrence is gradually increasing, risk factors remain a major concern and on the lookout as preventable measures (Yoon et al., 2009). Chronic cigarette smoking (CS) is a well‐known risk factor for various chronic diseases including neurological, cardiovascular, and pulmonary diseases (Das, 2003). More recently, several studies highlight the association of CS with an elevated risk of CKD onset and progression, independent of other well‐known risk factors such as age, body mass index, hypertension, diabetes, and NSAID usage (Fox et al., 2004; Haroun et al., 2003; Hippisley‐Cox & Coupland, 2010; Junior et al., 2014; Nagasawa, Yamamoto, Rakugi, & Isaka, 2012; Shankar, Klein, & Klein, 2006; Xia et al., 2017; Yoon et al., 2009). Both local and systemic effects following CS exposure are well known to promote organ injuries including the cardiovascular‐renal system by boosting both oxidative stress and inflammation (Hall et al., 2016; Jones‐Burton et al., 2007; Kaplan et al., 2017; Kobeissy et al., 2017; Mur et al., 2004; Noborisaka, Ishizaki, Yamazaki, Honda, & Yamada, 2014; Odoni et al., 2002; Ozbek, 2012; Rubanyi, 1988; Shiels et al., 2014; Toda & Toda, 2010). CS constitutes a risk factor for multiple diseases including diabetes, hypertension, metabolic syndrome, and atherosclerosis that ultimately fuel the cardiovascular–renal injury (Craig, Palomaki, & Haddow, 1989; Freedman et al., 1986; Guberina et al., 2013; Houston et al., 2006; Ishizaka et al., 2005; Nakamura et al., 2009; Ohkuma et al., 2015; Park, Oh, Cho, Choi, & Kim, 2004; Ueyama et al., 2017). Tobacco smoke contains thousands of compounds including cadmium and lead that are proven to be nephrotoxic (Hwangbo et al., 2011; Navas‐Acien et al., 2009). Nicotine itself is known to promote fibrosis and inflammation in the proximal tubule and induces apoptosis in podocytes (Dasgupta & Chellappan, 2006; Jaimes, Tian, Joshi, & Raij, 2009; Lan et al., 2016; Rezonzew et al., 2012). Although CS exposure time and amount highly correlate with CKD, heavy smokers remain at high risk of CKD for ten years following smoking cessation (Bleyer, Shemanski, Burke, Hansen, & Appel, 2000; Hallan & Orth, 2011; Jin, Koh, Chow, Yuan, & Jafar, 2013; Junior et al., 2014; Xia et al., 2017). Multiple clinical studies suggest that both males and females are susceptible to cardiovascular and kidney damage following CS exposure with premenopausal females being more protected (Baylis, 2012; Briganti et al., 2002; Garcia‐Esquinas, Loeffler, Weaver, Fadrowski, & Navas‐Acien, 2013; Guberina et al., 2013; Junior et al., 2014; Tozawa et al., 2002). To date, however, no experimental studies have been designed to thoroughly investigate gender‐biased CS‐induced kidney damage in a well‐controlled CS exposure environment.

Reactive oxygen species (ROS) and inflammation are interchangeable mechanisms that are both mediated by CS (Al‐Awaida, Akash, Aburubaiha, Talib, & Shehadeh, 2014; Das et al., 2012; Kaplan et al., 2017). Of note, the link between inflammation and kidney fibrosis has been well established and the pharmacological suppression of inflammation slows down renal dysfunction with a reduction in glomerulosclerosis and interstitial fibrosis (Beer, Mayer, & Kronbichler, 2016; Braun et al., 2008; Eardley et al., 2008). The disparity in inflammatory markers between genders is also established and mainly attributed to sex hormones and other gender‐biased factors such as body composition, physical/sexual maturation, and behavioral changes during adolescence (Mattina, Lieshout, & Steiner, 2019; Shanahan et al., 2013). The risk of chronic inflammation and the associated diseases is inversely proportional to aging during which sex hormones in both genders significantly decrease but to a different extent (Laaksonen et al., 2003; Mattina et al., 2019). Women smokers are now known to be at higher risk of developing dyslipidemia, cardiovascular diseases, and lung diseases (Hsu et al., 2017; Lee et al., 2011; Peters, Huxley, & Woodward, 2014; Yahagi, Davis, Arbustini, & Virmani, 2015). This study is the first to provide experimental evidence of gender‐biased kidney damage in CS‐exposed mice highlighting therefore the importance of sex hormones in pathophysiological responses.

## 2 MATERIALS AND METHODS

### 2.1 Animals

All animal experiments were carried out according to an experimental protocol approved by the Institutional Animal Care and Use Committee in compliance with the Guide for Care and Use of Laboratory Animals of the Institute for Laboratory Animal Research of the National Academy of Sciences, USA, and the animal facility at the American University of Beirut Medical Center, Lebanon.

### 2.2 Experimental design

This study was performed with C57BL/6J mice, which are the most widely used mouse model of human diseases involving genetic modifications, and which exhibit disease‐related oxidative stress profiles comparable to humans. Six‐month‐old C57BL/6J mice (equivalent to 30 years old human age) were allocated into four groups: CS‐exposed male, non‐CS‐exposed male, CS‐exposed female, and non‐CS‐exposed female. Male and female gender weight ranged between 25–30 and 20–25 g, respectively. Male and female CS‐exposed groups underwent the same CS exposure protocol in the same period and time. For kidney collection, each mouse was placed in an isoflurane induction box for 30 s to 1 min until complete unconsciousness. Mouse was then placed on the surgical board with maintained unconsciousness under 2%–3% isoflurane anesthesia using an oxygen‐based isoflurane vaporizer. Anesthetic depth was assessed by toe pinch before any surgical procedure. Right kidney was harvested, crushed and split in half for Western blotting and PCR analysis. Each half was then immediately placed into a cryotube in liquid nitrogen followed by storage at −80°C. Left kidney was harvested into 10% zinc formalin tubes for histology. Following kidney collection, anesthesia was increased to 5% for 1 min followed by cervical dislocation of the animal. All measurements and scoring were performed blindly.

### 2.3 Echocardiography

Transthoracic echocardiography was performed 6 weeks after smoke exposure protocol by using SonixTouch ultrasound system (Ultrasonix Medical Corporation) equipped with a high‐frequency linear array transducer. Mice were anesthetized with 1%–2% isoflurane in an oxygen mix and placed on a heated electrical platform. B‐mode and M‐Mode images of left ventricular were acquired from the parasternal long‐axis view in a supine position. Body temperature, heart rate, and respiratory rate were continuously monitored throughout the procedure. M‐mode measurements of the left ventricle included the following: left ventricular end‐diastolic diameter (LVEDD) and left ventricular end‐systolic diameter (LVESD) normalized to body weight. Left ventricular fractional shortening (FS) was calculated according to the following formula: FS (%) = (LVEDD − LVESD/LVEDD) x 100.

### 2.4 Cigarette smoking protocol

Briefly, age‐matched male and female C57BL/6J mice were exposed to cigarette smoke using a cigarette smoking exposure apparatus (ONARES, CH Technologies). This apparatus includes a smoke generator with a mixing/conditioning chamber and a “nose‐only” rodent exposure carousel. It allows for exposure to mainstream smoke from a cigarette in conscious, restrained rodents. This system has been extensively used to study smoking‐related diseases. Mice in the smoking groups were exposed to the smoke of 3R4F research‐grade cigarettes (University of Kentucky), which are scientifically prepared cigarettes concentrated with toxins and chemicals rendering the study timeline suitable to observe the effects of smoking on the mice. Cigarettes were placed into the cigarette puffer, and a peristaltic pump was used to generate puffs at a frequency of 1 puff/min, duration of 2.5 s/puff, and a volume of 5 ml/puff. Mice received two 60‐min CS sessions per day (7 days/week) for 6 weeks allowing a total particular matter (TPM) concentration of about 100 mg/cm^3^/mouse/session, 9.4 mg tar, and 0.726 mg nicotine per cigarette. The amount of TPM was measured by weighing the smoke filters before and after the experiment. CS exposure in each session was adjusted according to mouse body weight.

### 2.5 Western blot

Snap‐frozen kidneys were ground in liquid nitrogen by a mortar and pestle and then homogenized in lysis buffer (1X RIPA buffer) containing protease and phosphatase inhibitors. Total protein concentrations were then determined using (DCTM Protein Assay II kit) Bradford/Lowry method (Bio‐Rad, Hercules). Protein samples (30 μg) were loaded into the wells of 12% SDS‐PAGE gel. Gels were then transferred to PVDF membranes at 4°C at 30–40 volts overnight. The membrane was then blocked in 5% fat‐free milk, prepared in 0.1% Tween PBS, for 1 hr at RT. Proteins were detected by primary monoclonal antibodies against interleukin 10 (IL‐10), interleukin 4 (IL‐4), interleukin 13 (IL‐13), interleukin 1 beta (IL‐1β), transforming growth factor beta (TGF‐β), tumor necrosis factor alpha (TNF‐α), and glyceraldehyde 3‐phosphate dehydrogenase (GAPDH) (Sigma); the latter was used to ensure equal loading of samples. Immunoblots were then probed with the appropriate secondary antibody (anti‐mouse 1:5,000, anti‐rabbit 1:10,000) for 1 hr at RT. Bands were detected with ECL chemiluminescence kit (Thermo Fisher Scientific). The intensity of bands was then determined by densitometry, using the ImageJ software (https://imagej.nih.gov/ij/).

### 2.6 Histopathology

Table 1 lists the type of stain used, number of animals, fields per animal, and subjects per field.

**Table 1:** List the type of stain used, test, number of animals, fields per animal, and subjects per field

Stain	Test	CS animals	Fields/animal	Subjects/animals
PAS	Glomerular pathology	3–6	10	>50 Glomeruli
PAS	Tubular pathology	3–6	10	>100 Tubules
Masson trichrome	Renal fibrosis	5–6	20	N/A
DHE	Glomerular/total ROS	3–6	10	>50 Glomeruli

#### 2.6.1 Periodic acid Schiff (PAS) staining

Paraffin‐embedded blocks were sectioned at 5 µm thicknesses. Five‐micrometer‐thick sections from each mouse were stained with PAS according to standard laboratory protocol. Briefly, after dewaxing and hydration steps of the paraffin‐embedded kidneys, the tissue was incubated with 0.5% periodic acid for 10 min followed by the Schiff reagent for 10–20 min and then washed for 5 min, according to manufacturer's protocol (Periodic Acid Schiff Stain Kit, Mucin Stain, Abcam, ab150680). Various steps of dehydration with an increased percentage of ethanol (75%, 95%, and 100%) were done, followed by clearing using xylol, and finally, slides were mounted using a mounting medium. Slides were then observed using light microscopy for the assessment of glomerular and tubular size.

#### 2.6.2 DHE staining

The levels of total and glomeruli reactive oxygen species (ROS) were assessed using dihydroethidium (DHE) staining (Calbiochem). Briefly, dewaxing and hydration steps were performed on formalin‐fixed paraffin‐embedded kidneys tissue sections. 10 µM DHE was used to incubate unstained slides in a humidified chamber under a dark condition at room temperature for 1 hr. Total and glomeruli ROS images were acquired using Scanning Fluorescent Microscope (Zeiss Axio) under 20× magnification; then, ImageJ software (https://imagej.nih.gov/ij/) was used to measure ROS score. For glomeruli ROS score, no specific glomerulus staining was applied. A visual identification of the glomeruli was performed to differentiate glomeruli from proximal tubules followed by threshold modification using Scanning Fluorescent Microscope (Zeiss Axio) in order to omit nonglomerulus ROS signals.

#### 2.6.3 Masson trichrome (MTC)

Renal fibrosis percentage was assessed using MTC staining. Dewaxing and hydration steps were performed on formalin‐fixed paraffin‐embedded kidney tissue sections. Kidney sections were then soaked in Bouin solution at 56°C for 1 hr, washed, and rinsed in distilled water. After 10‐min incubation with hematoxylin, kidney tissues were washed and stained in biebrich scarlet acid fuchsin. A second wash was performed, and kidney sections were differentiated in phosphomolybdic–phosphotungstic acid solution for 10 min, transferred to aniline blue solution, stained for 5 min, and observed under light microscopy at 10× magnification. ImageJ software (https://imagej.nih.gov/ij/) was used to measure renal fibrosis. Representative figures were taken under 20× magnification.

#### 2.6.4 Calculation

The slides stained with PAS were used to assess the glomerular cross‐sectional area (CSA), glomerular tuft area, Bowman's space, and proximal tubule and lumen diameter. At least three kidney sections per mouse of each group underwent evaluation. Ten fields of PAS‐stained renal sections from each mouse were selected under light microscopy at 20× magnification. More than 50 glomeruli per group were analyzed. Glomeruli that were sectioned at midpoint with commonly visible vascular pole and afferent and efferent arterioles were included in the analysis. The ones that were sectioned close to the pole of the glomerular sphere and had deformation in shape and borders were excluded. Glomerular and glomerular tuft cross‐sectional areas were computed using ImageJ software version 1.42 (National Institutes of Health). Bowman's space was determined by subtracting the glomerular area from the glomerular tuft area. More than 100 symmetrically sectioned proximal tubules were selected to assess tubular diameter and tubular lumen diameter. A similar method with previous studies was used for assessment (Leh, Hultstrom, Rosenberger, & Iversen, 2011; Rangan, Wang, Tay, & Harris, 1999). Proximal tubular diameter was defined as the distance between two points on the basal membrane of the tubule connected by the shortest straight line that passes through the center of a tubule. Proximal tubule lumen diameter was defined as the distance between the luminal surfaces of two cells on the straight line.

### 2.7 Statistical analysis

All experiments were performed for an *n* of six animals or as otherwise indicated. Results were expressed as fold change or mean ± the standard error of the mean (*SEM*). Statistical comparisons were performed using an unpaired or paired *t* test followed by Mann–Whitney and Wilcoxon nonparametric tests, respectively, for non‐Gaussian distributions. The *p*‐value of *p* < .05 (*), *p* < .01(**), and *p* < .001(***) was considered significant. GraphPad Prism 5 software was used to perform statistical analysis.

## 3 RESULTS

### 3.1 Significant variation in body weight of both genders following CS exposure

**Figure 1:**
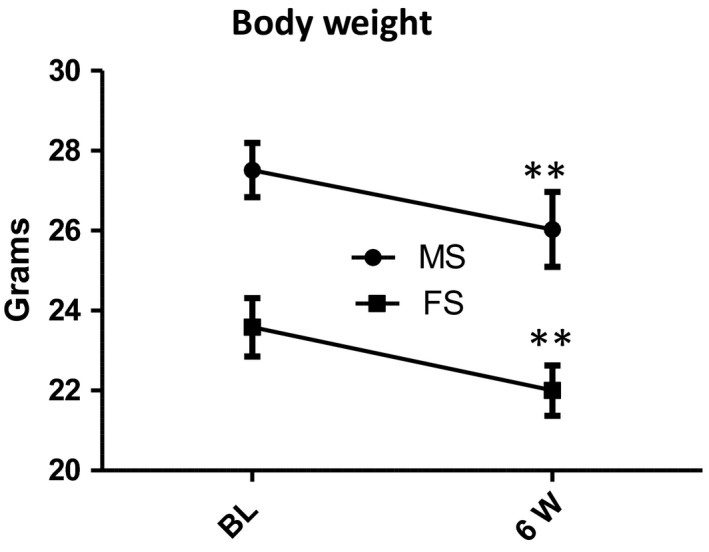
CS significantly decreases body weight in both genders. Body weight of both male and female mice significantly decreased following 6 weeks of cigarette smoke exposure when compared to baseline levels. Paired *t* test revealed the significance before and after CS exposure of each gender. All bars represent mean ± *SEM* (**p < .01; *n* = 6). FS, female smoking; MS, male smoking

CS‐exposed mice of both genders experienced a significant decrease in body weight (BW) at week 6 following CS exposure (Figure 1). These findings confirm the well‐known anorexic effects of CS (Audrain‐McGovern & Benowitz, 2011) and highlight the successful exposure of the animals to CS. BW at baseline and week 6 of CS‐exposed male mice was 27.52 ± 0.6 and 26.03 ± 0.9 g, respectively (**p < .01). In female mice, BW at baseline and following 6 weeks of CS exposure was 23.58 ± 0.7 and 22 ± 0.6 g, respectively (**p < .01).

### 3.2 Both genders experienced no change in cardiac fractional shortening following CS exposure

**Figure 2:**
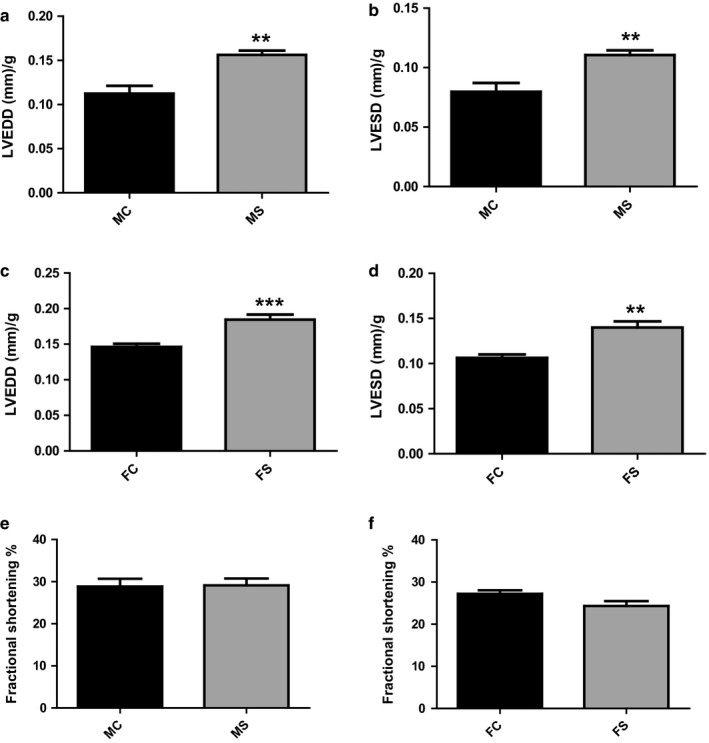
No effect of CS on cardiac systolic hemodynamics in both genders. LVEDD and LVESD in both male and female mice significantly increased following 6 weeks of CS exposure when compared to their relative control groups (a–d). LV fractional shortening (FS) in both male (e) and female (f) mice was not significant when compared to relative control groups following 6 weeks of CS exposure. Data were analyzed for significance using unpaired *t* test. All bars represent mean ± *SEM* (*n* = 6) (**p < .01;***p < .001). FC, female control; FS, female smoking; MC, male control; MS, male smoking

No significant change was seen with FS in both CS‐exposed genders when compared to their relative controls (Figure 2). As detailed in the method section of the manuscript, left ventricular FS was calculated according to the following formula: FS (%) = (LVEDD − LVESD/LVEDD) × 100. In fact, both LVEDD and LVESD parameters of both genders significantly but proportionally increased following CS exposure resulting therefore in a nonsignificant change in FS levels when compared to the relative control groups (Figure 2a–d). FS at week 6 of CS‐exposed and non‐CS‐exposed male mice was 29.1% and 28.9%, respectively (Figure 2e). Female mice, on the other hand, had inherently lower FS than male mice; FS at week 6 of CS‐exposed and non‐CS‐exposed female mice was 27.2% and 24.3%, respectively (Figure 2f).

### 3.3 Glomerular oxidative stress increased in both genders following CS exposure

**Figure 3:**
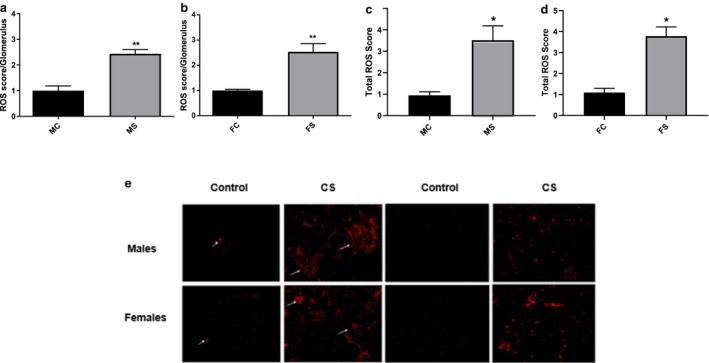
Equivalent ROS increase in both genders following CS. Glomerular ROS (a and b) and total ROS (c and d) significantly increased to comparable levels in both male (a and c) and female (b and d) mice following 6 weeks of CS exposure when compared to relative baseline groups as seen with DHE staining. Representative figure of glomerular ROS score in each group is shown (e). Data were analyzed for significance using unpaired *t* test. All bars represent fold change ±*SEM* (**p < .01. *p < .05). FC, female control (*n* = 3); FS, female smoking (*n* = 6); MC, male control (*n* = 3); MS, male smoking (*n* = 5)

CS‐exposed mice of both genders experienced a significant increase in glomerular oxidative stress following 6 weeks of CS exposure (Figure 3a). Glomerular ROS and total ROS production at week 6 of CS‐exposed male significantly increased by 2.36 ± 0.24‐fold and 3.15 ± 0.63‐fold, respectively, when compared to non‐CS‐exposed male group (**p < .01) (Figure 3a). Similarly, female mouse glomerular ROS and total ROS following 6 weeks of CS exposure significantly increased by 2.53 ± 0.32‐fold and 3.78 ± 0.44‐fold, respectively, when compared to non‐CS‐exposed female group (**p < .01) (Figure 3b).

### 3.4 CS‐exposed mice showed gender‐biased differences with renal inflammatory and fibrotic cytokine levels

**Figure 4:**
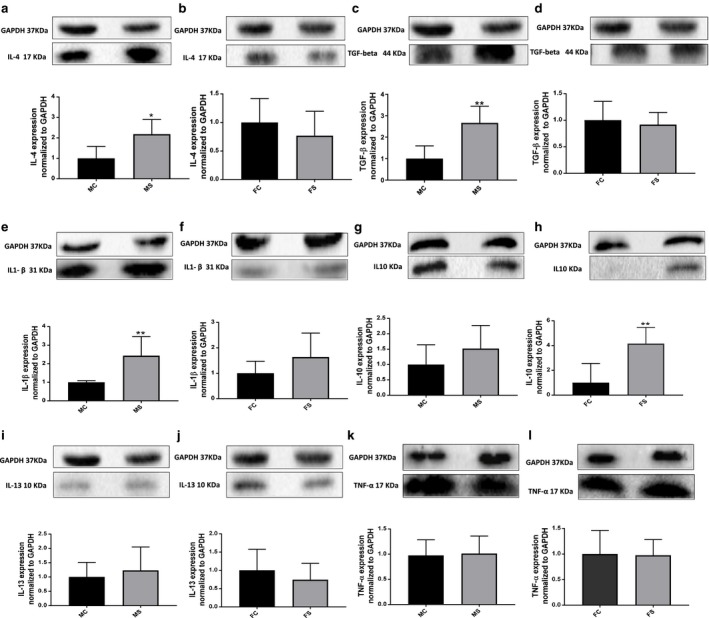
Higher inflammatory and fibrotic markers in CS‐exposed male group. IL‐4 levels significantly increased in male (a) but not in female (b) mice when compared to their relative control groups, 6 weeks following CS exposure. Similar pattern is observed with TGF‐β and IL‐1β levels (c–f). IL‐10 on the other hand was unchanged in male (g) but significantly increased in female (h) mice when compared to their relative control groups and following CS exposure. No significant difference was observed with IL‐13 and TNF‐α in male and female CS‐exposed mice when compared to their relative control groups (i–l). Representative image of each blot is shown. Data were analyzed for significance using unpaired *t* test. All bars represent fold change ±*SEM* after normalization to GAPDH (*p < .05; **p < .01; *n* = 5–6). FC, female control; FS; female smoking; MC, male control; MS, male smoking; IL, interleukin; GAPDH, glyceraldehyde 3‐phosphate dehydrogenase as housekeeping protein; TNF‐α, tumor necrosis factor alpha; TGF‐β, transforming growth factor beta

Western blot analysis revealed a significant increase in IL‐4 protein levels in the kidneys of CS‐exposed male mice when compared to their relative control (2.1‐fold change, *p* < .05) (Figure 4a). Female mice, however, of both CS and non‐CS‐exposed groups exhibited no difference in IL‐4 protein levels (Figure 4b). Similarly, TGF‐β1 protein levels were significantly high in CS‐exposed male mice when compared to their relative control (2.6‐fold change, **p < .01), whereas no significant change was observed between female mice of both groups (Figure 4c and d). IL‐1β reached significance only in CS‐exposed male group when compared to the non‐CS‐exposed male group (2.4‐fold change, **p < .01) with no significant change observed between female groups (Figure 4e and f). On the other hand, IL‐10 protein levels reached significance only in the CS‐exposed female group when compared to non‐CS‐exposed female group (4.1‐fold change, **p < .01), with no significant change between male groups (Figure 4g and h). IL‐13 and TNF‐α protein expression did not change significantly between both the CS‐exposed and the non‐CS‐exposed male and female groups (Figure 4i–m).

### 3.5 CS‐exposed mice exhibited biased gender differences with histological markers

#### 3.5.1 Renal fibrosis

**Figure 5:**
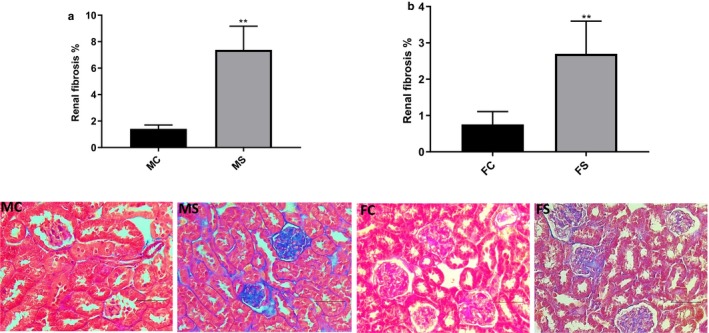
Renal fibrosis percentage significantly increased in both genders. Male (a) and female (b) mouse renal fibrosis percentage following 6 weeks of CS exposure when compared to relative baseline groups. Representative image of each Masson trichrome‐stained group is shown. Data were analyzed for significance using unpaired *t* test. All bars represent fold change ±*SEM* (**p < .01; *n* = 5–6). Scale bars = 100 µm. FC, female control; FS, female smoking; MC, male control; MS, male smoking

Masson trichrome staining revealed enhanced renal fibrosis percentage in CS‐exposed groups of both genders, with CS‐exposed male group exhibiting 5.2‐fold increase versus 3.5‐fold increase in CS‐exposed female group when compared to their relative control group (**p < .01) (Figure 5a and b).

#### 3.5.2 Glomerular pathology

**Figure 6:**
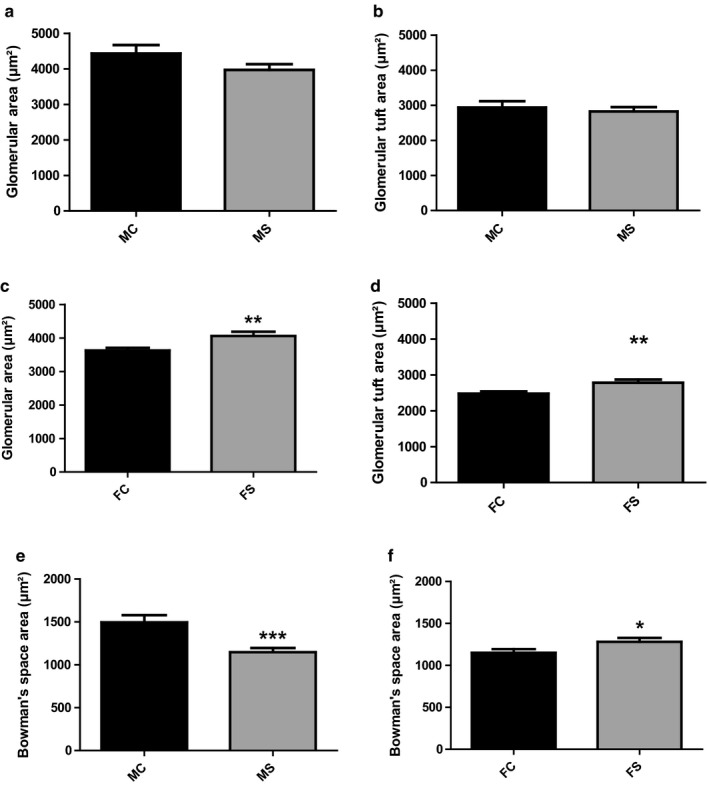
Signs of glomerular atrophy in CS‐exposed male mice. Quantitative assessment of glomerular structure changes in both male and female mice following CS exposure and PAS staining. Unlike CS‐exposed male mice that showed no significant changes (a and b), glomerular CSA, and glomerular tuft CSA significantly increase in CS‐exposed female mice groups when compared to their relative control groups (c and d). The Bowman space area significantly decreased in CS‐exposed male mice group (e), whereas a significant increase was observed in CS‐exposed female mice group when compared to their relative control groups (f). Data were analyzed for significance using unpaired *t* test. All bars represent mean ± *SEM* (*p < .05, **p < .01; ***p < .001 *n* = 3–6). FC, female control; FS, female smoking; MC, male control; MS, male smoking; CSA, cross‐sectional area

Glomerular pathology was assessed by PAS staining. Glomerular and glomerular tuft CSA were both insignificantly smaller in CS‐exposed male mice when compared to their relative control group with 3,968 ± 162.7 *N* = 79, 4,432 ± 235.4 *N* = 52, respectively, for glomerular CSA and 2,821 ± 127.2 *N* = 79, 2,936 ± 177.6 *N* = 52, respectively, for glomerular tuft CSA (Figure 6a and b). In contrast, both glomerular CSA and glomerular tuft CSA were significantly greater in CS‐exposed female mice when compared to their relative control group with 4,062 ± 121.0 *N* = 74, 3,628 ± 80.28 *N* = 91, respectively, **p < .01, for glomerular CSA; and 42,782 ± 86.94 *N* = 74, 2,477 ± 61.93 *N* = 91, respectively, **p < .01, for glomerular tuft CSA (Figure 6)c and d). Bowman's space area was significantly narrower in CS‐exposed male mice when compared to their relative control group (1,147 ± 48.21 *N* = 79, 1,495 ± 84.74 *N* = 52, respectively, ***p < .001) (Figure 6e). In contrast, CS‐exposed female mice exhibited a significant increase in Bowman's space area when compared to non‐CS‐exposed female group (1,280 ± 47.48 *N* = 74, 1,150 ± 43.36 *N* = 91, respectively, *p < .05) (Figure 6f). *N* = subjects per field.

#### 3.5.3 Tubular pathology

**Figure 7:**
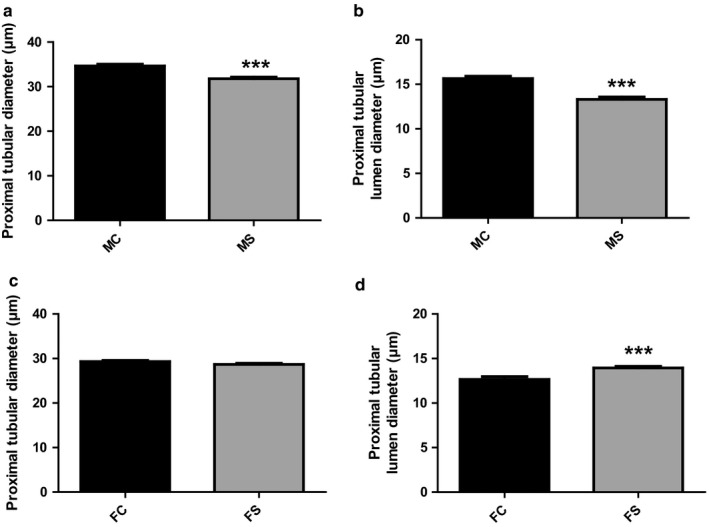
Signs of tubular atrophy in CS‐exposed male mice. Quantitative assessment of tubular structure changes in both male and female mice following CS exposure and PAS staining. Both proximal tubular and proximal tubular lumen diameter significantly decreased in CS‐exposed male mice when compared to their relative control groups (a and b). In female mice, however, no significant change was observed in proximal tubular diameter between groups (c). However, a significant increase in proximal tubular lumen diameter in CS‐exposed female group was observed when compared to the relative control group (d). Data were analyzed for significance using unpaired *t* test. All bars represent mean ± *SEM* (***p < .001 *n* = 3–6). FC, female control; FS, female smoking; MC, male control; MS, male smoking

Tubular pathology was assessed via PAS staining. Both Proximal tubular diameter and proximal tubular lumen diameter were significantly smaller in CS‐exposed male mice when compared to their relative control group (31.79 ± 0.34 *N* = 106, 34.66 ± 0.39 *N* = 143, respectively, ***p < .001) and (13.31 ± 0.25 *N* = 106, 15.64 ± 0.26 *N* = 143, respectively, ***p < .001), respectively (Figure 7a and b). In female group, proximal tubular diameter was similar between CS‐exposed and non‐CS‐exposed female groups (28.68 ± 0.27 *N* = 137, 29.32 ± 0.28 *N* = 146, respectively). However, proximal tubular lumen diameter significantly increased in CS‐exposed female group when compared to their relative control group (13.94 ± 0.18 *N* = 137, 12.66 ± 0.32 *N* = 145, respectively, ***p = .001) (Figure 7)c and d). *N* = subjects per field.

## 4 DISCUSSION

This study reports the differential impact of CS on kidney damage between genders. Kidney damage was assessed at both molecular and structural levels in CS‐exposed male and female mice. Increased inflammation, oxidative stress, fibrosis, and structural modification findings highly correlated with CS exposure in both genders but to a different extent. For instance, CS‐exposed male mice suffered advanced renal inflammation, increased fibrosis, and worsened structural changes than age‐matched CS‐exposed female mice when compared to their relative control groups. CS‐induced injury is majorly attributed to chronic oxidative stress bursts due to cigarette compounds that are either oxidants, pro‐oxidants, or alter the cellular antioxidant battery (Al‐Awaida et al., 2014; Aoshiba & Nagai, 2003; Devasagayam et al., 2004; Husain, Scott, Reddy, & Somani, 2001; Kaplan et al., 2017). Nicotine itself is known to upregulate oxidative enzymes in the kidneys through binding to α7‐nAChR subunit of renal nicotinic receptors (Rezonzew et al., 2012). Our study revealed a comparable renal ROS increase in both genders following 6 weeks of CS exposure when compared to their relative control group. This finding suggests that the renoprotective effects observed in female mice are not due to direct antioxidant mechanisms but rather to a protection downstream of ROS‐induced injury. This assumption is fortified with our inflammatory and fibrotic marker findings including IL‐1β, IL‐4, IL‐10, and TGF‐β levels. Of note, inflammation is directly implicated in the early stages of kidney pathogenesis and constitutes the hallmark of ROS‐mediated harmful effects (Al‐Awaida et al., 2014; Cottone et al., 2009; Hall et al., 2016; Kantengwa, Jornot, Devenoges, & Nicod, 2003; Nerpin et al., 2012; Noborisaka et al., 2014; Oberg et al., 2004; Rodriguez‐Iturbe & Garcia, 2010). Multiple studies associated high IL‐1β levels with CS exposure in different tissues (Doz et al., 2008; Ebersole, Steffen, Thomas, & Al‐Sabbagh, 2014; Pauwels et al., 2011; Shiels et al., 2014). Our data showed that CS exposure significantly increased renal proinflammatory IL‐1β levels in male, but not in female mice when compared to their relative control groups. TNF‐α, a master regulator of inflammation with a critical role in the initiation, maintenance, and/or progression of inflammation, was unchanged in both genders following 6 weeks of CS exposure. These findings correlate and contradict with multiple basic and clinical studies with respect to the organ studied, duration of CS exposure, the amount of cigarette smoked per day, and the presence or absence of comorbidities (Feng et al., 2013; Machado et al., 2018; Mizia‐Stec, Zahorska‐Markiewicz, & Gasior, 2004; Parameswaran & Patial, 2010; Petrescu, Voican, & Silosi, 2010; Szulinska et al., 2013; Verschuere et al., 2011). Inflammation is generally associated with an anti‐inflammatory (i.e., IL‐10, IL‐13) and profibrotic (i.e., TGF‐β) response depending on the severity and the duration of inflammation. A compromised anti‐inflammatory response, however, could prolong inflammation and exacerbate therefore fibrotic deposition. In that regard, increased renal IL‐10 levels to a significant extent were only observed in female mice of our study, highlighting the gender‐biased protective anti‐inflammatory mechanisms following CS exposure. Of note, a considerable number of experimental and clinical studies reported either a decreased (Arnson, Shoenfeld, & Amital, 2010; Li, Wang, Cao, Ma, & Xu, 2015; Nakamura et al., 2014; Ugur, Kutlu, & Kilinc, 2018), increased (Chen et al., 2013; Lim et al., 2000), or unchanged levels (Mizia‐Stec et al., 2004; Zeidel et al., 2002) of IL‐10 following CS exposure which was directly linked to the type of species, type of tissues, level of smoking, and time points studied (Arnson et al., 2010; Chen et al., 2013; Li et al., 2015; Lim et al., 2000; Mizia‐Stec et al., 2004; Nakamura et al., 2014; Ugur et al., 2018; Zeidel et al., 2002). There is a consensus, however, that IL‐10 plays a significant role in reducing the inflammatory response and preventing myofibroblast proliferation and collagen deposition in multiple renal pathologies (Sziksz et al., 2015). IL‐13, on the other hand, exhibited comparable renal levels in both genders when compared to their relative control groups. IL‐13 is known for its anti‐inflammatory, antiapoptotic, and antioxidant properties that could have explained, if significantly increased, the protection observed in CS‐exposed female mice (Evans & Kilshaw, 2000; Kato, Okaya, & Lentsch, 2003; Minty et al., 1993). However, these findings are not surprising given that IL‐13 is known to fluctuate following CS exposure between high, low, or unchanged levels depending on the disease state, tissue studied, and other parameters (Jafarzadeh, Esmaeeli‐Nadimi, Nough, Nemati, & Rezayati, 2009; MalekZadeh, Nikbakht, Sadeghi, Singh, & Sobti, 2010). With unchanged IL‐10 and IL‐13 levels, CS‐exposed male mice showed a significant increase in IL‐4 and TGF‐β levels that remained unchanged in CS‐exposed female mice. IL‐4 is a pleiotropic cytokine engaged in several pathways that range from a classical anti‐inflammatory action with type 2 like effect to TGF‐β upregulation and fibrosis formation independently or in combination with multiple other processes (Luzina et al., 2012; Pardo & Selman, 2002; Sempowski, Beckmann, Derdak, & Phipps, 1994; Sime & O'Reilly, 2001; Striz et al., 1999). Interestingly, both experimental and clinical studies are in accordance with our findings and documented an increase in IL‐4 levels in serum and pulmonary tissue following CS exposure with a significance only seen in CS‐exposed men (Byron, Varigos, & Wootton, 1994; Flouris et al., 2009; Kemeny et al., 2017; Matsunaga et al., 2014; Nakamura et al., 2014; Sahlander, Larsson, & Palmberg, 2010; Zhu et al., 2001). TGF‐β1 is also a pleiotropic and multifunctional cytokine with profibrotic actions and is known to be upregulated in the kidneys of CS‐exposed subjects and plays a major role in the development of progressive glomerulosclerosis and interstitial fibrosis (Elliot et al., 2006; Mur et al., 2004). The observed cytokine pattern following CS exposure in this study could explain the pathophysiological differences observed between genders. For instance, cytokine patterns following CS exposure in male mice resemble a prevailing innate‐based inflammatory profile (IL‐1β) along with an adaptive Th2‐like response characterized by an increase in IL‐4 levels and translated into pronounced inflammation and fibrosis. Female CS mice, on the other hand, exhibited a low‐level inflammatory and fibrotic profile characterized by a significant increase in the anti‐inflammatory cytokine IL‐10, suggesting a potential participation of protective regulatory T cells in this pathophysiology. Of note, estrogen is known to promote regulatory T‐cell expansion and activation and whether it is directly involved in this process warrants future investigation (Polanczyk et al., 2004; Tamosiuniene et al., 2018). The proinflammatory and profibrotic markers analyzed in this study are in line with our histological findings. Renal fibrosis significantly increased in both CS‐exposed groups, but to a greater extent in the CS‐exposed male group, when compared to their relative controls. This concludes that both genders are prone to injury following comparable CS‐induced ROS burst relative to their control groups but to a lesser extent in the female group. These findings could be explained by the fact that female mice were able to better control ROS‐mediated inflammatory response than male mice and therefore lessened CS‐induced renal damage. In fact, multiple studies support the concept of estrogen antioxidant capacity, and their reduction in postmenopausal women highly correlates with increased ROS levels (Kim, Pedram, Razandi, & Levin, 2006; Lejskova, Alusik, Valenta, Adamkova, & Pitha, 2012; Szabo et al., 2018; Yung et al., 2011). To further assess kidney damage, glomerular hypertrophy, a marker of nephron loss and early kidney diseases, was assessed (Brenner, 1985; Hattori, Kim, Steffes, & Mauer, 1993). Glomerular CSA, tuft area, and Bowman's space area were significantly increased in the CS‐exposed female group only. Glomerular hypertrophy generally represents a marked hyperfiltration, increased intraglomerular pressure, and increased dilatation of Bowman's space area as a sign of early kidney injury that usually preludes glomerular collapse and loss (Brenner, 1985; Chagnac et al., 2000; Kambham, Markowitz, Valeri, Lin, & D'Agati, 2001; Maeda et al., 2011; Mogensen, Christensen, & Vittinghus, 1983; Puelles et al., 2014). In CS‐exposed male group, however, glomerular hypertrophy was absent with a significant decrease in Bowman's space area, suggestive of potential glomerular collapse and an advanced stage of kidney damage than CS‐exposed female group. Multiple studies have documented the direct effect of CS and nicotine on intraglomerular pressure, glomerular hyperfiltration, and Bowman's space dilatation and are in line with our general observation (Guberina et al., 2013; Hammer, Cohen, Levi, & Krause, 2016; Jin et al., 2013; Maeda et al., 2011; Noborisaka, 2013; Popa et al., 2017; Tobar et al., 2013; Yoon et al., 2009). Proximal tubular dilatation was also assessed in this study given its link to glomerular hyperfiltration and the fact that tubular epithelial cells express nAChR subunits in addition to their sensitivity to toxic metabolites (Arany, Carter, Hall, Fulop, & Dixit, 2017; Kim et al., 2016; Tobar et al., 2013). CS‐exposed female mice exhibited a significant increase in proximal tubular lumen diameter but not total tubular diameter which is in accordance with mild glomerular hyperfiltration effects and early stages of kidney injury (Tobar et al., 2013). CS‐exposed male mice, however, presented with a significant decrease in both tubular and tubular lumen diameter. Of note, reduced tubular diameter consistent with chronic tubular atrophy is considered a superior predictor of CKD than glomerular pathology and is generally associated with glomerular hypoperfusion and collapse (Leh et al., 2011; Schelling, 2016). Decrease in Bowman's space and reduced tubular diameter observed in CS‐exposed male mice highlight therefore the advanced stage of kidney damage that was not observed in the CS‐exposed female group.

Nonrenal parameters were also assessed in this study including body weight and cardiac systolic function. The lowering impact of CS on body weight in both genders was expected and documented in multiple studies given the hypophagia features of nicotine and other conventional cigarette compounds as well as their impact on metabolic rate (Bishop, Parker, & Coscina, 2004; Jitnarin et al., 2014; Talukder et al., 2011; Wack & Rodin, 1982). At the cardiac level, CS is known to exert direct and indirect adverse cardiac effects including cardiac remodeling (Gu, Pandey, Geenen, Chowdhury, & Piano, 2008; Kaplan et al., 2017). In this study, 6 weeks of CS exposure increased LVEDD and LVESD significantly, but proportionally, of both genders resulting therefore in an unchanged left ventricular FS and cardiac function. These results suggest that the observed renal injury is potentially due to direct CS effects rather than CS‐induced cardiac systolic dysfunction and subsequent systemic hemodynamic alteration that is usually observed with longer CS exposure time (Talukder et al., 2011). This finding emphasizes the importance of renal damage as a forerunner and an early contributor to CS‐induced cardiovascular adverse effects.

## 5 CONCLUSION AND FUTURE DIRECTIONS

The outcome of this study suggests that CS‐exposed males are more prone to early kidney disease development than CS‐exposed female mice. However, these findings are not surprising given the well‐documented estrogen protection of multiple systems in the body including the cardiovascular and renal systems (Morselli et al., 2017). In fact, multiple preclinical studies have documented estrogen protection in different forms of kidney injury including ischemia‐reperfusion injury, kidney transplantation, cardiac arrest/cardiopulmonary resuscitation, and ER stress‐induced injury (Pan & Sheikh‐Hamad, 2019). Elimination of estrogen protection by ovariectomy was restored by estrogen administration. In our study, the protection observed in females seems to be mediated downstream of ROS production given that both genders exhibited the same levels of CS‐induced ROS production when compared to their relative controls. This finding was surprising given that multiple studies highlight differences in ROS generation between genders under several chronic conditions such as hypertension (Kafami et al., 2018; Lopez‐Ruiz, Sartori‐Valinotti, Yanes, Iliescu, & Reckelhoff, 2008), diabetes (Kotani, Tsuzaki, Taniguchi, & Sakane, 2013), and perinatal iron deficiency with high salt diet (Woodman et al., 2020). Additionally, it is now believed that sex hormone controls ROS production, in part, through antioxidant gene expression (Bellanti et al., 2013), potentially due to sex differences in mitochondrial biology and tissue and age variability (Pan & Sheikh‐Hamad, 2019). However, normal mice were implemented in this study and CS itself is rich in exogenous ROS compounds that might explain the equivalent renal ROS levels observed following CS exposure. In addition to comparable ROS levels, both genders were characterized by a significant increase in renal fibrosis and glomerulotubular alteration, with males, however, exhibiting an advanced stage of renal damage with more pronounced inflammation and fibrosis. Future experiments deciphering the actual pathways behind those findings are warranted. For instance, several studies revealed that estrogen possesses antifibrotic properties through modulating TGF‐β levels that were preserved in our CS‐exposed female mice (Blush et al., 2004; Karl, Berho, Pignac‐Kobinger, Striker, & Elliot, 2006; Maric, Sandberg, & Hinojosa‐Laborde, 2004). Finally, although protection in CS‐exposed females seems to prevail in the early stage of CS exposure, the parameters observed indicate that both genders are on the slope of CKD development. In fact, CS compounds are now known to exert antiestrogenic properties and increased androgens‐to‐estrogen ratio throughout a CS life that can alter, in the long run, estrogen protective effects (Jandikova, Duskova, & Starka, 2017; Tanko & Christiansen, 2004).

## CONFLICT OF INTEREST

The authors declare no competing financial interests and therefore have nothing to disclose.

## Data Availability

The data that support the findings of this study are available from the corresponding author upon reasonable request.
